# Cystatin C serum levels in healthy children are related to age, gender, and pubertal stage

**DOI:** 10.1007/s00467-018-4087-z

**Published:** 2018-11-20

**Authors:** Niels Ziegelasch, Mandy Vogel, Eva Müller, Nadin Tremel, Anne Jurkutat, Markus Löffler, Nicolas Terliesner, Joachim Thiery, Anja Willenberg, Wieland Kiess, Katalin Dittrich

**Affiliations:** 1 LIFE Leipzig Research Center for Civilization Diseases, University of Leipzig, Philipp-Rosenthal-Strasse 27, 04103 Leipzig, Germany; 2 Center of Paediatric Research (CPL), University of Leipzig, 04103 Leipzig, Germany; 3 Institute for Medical Informatics, Statistics and Epidemiology (IMISE), University of Leipzig, 04107 Leipzig, Germany; 4 Hospital for Children and Adolescents, University of Leipzig, Liebigstraße 20a, 04103 Leipzig, Germany; 5 Institute for Laboratory Medicine, Clinical Chemistry and Molecular Diagnostics (ILM), University Hospital Leipzig, 04103 Leipzig, Germany

**Keywords:** Children, Adolescents, Cystatin C, Serum creatinine, Reference values

## Abstract

**Background:**

This study aims to establish age- and gender-specific cystatin C (CysC) reference values for healthy infants, children, and adolescents and to relate them to pubertal stage, height, weight, and body mass index (BMI).

**Methods:**

Serum CysC and creatinine levels of 6217 fasting, morning venous blood samples from 2803 healthy participants of the LIFE Child study (age 3 months to 18 years) were analyzed by an immunoassay. Recruitment started in 2011; 1636 participants provided at least one follow-up measurement. Percentiles for CysC were calculated. Age- and gender-related effects of height, weight, BMI, and puberty status were assessed through linear regression models.

**Results:**

Over the first 2 years of life, median CysC levels decrease depending on height (*ß* = − 0.010 mg/l/cm, *p* < 0.001) and weight (*ß* = − 0.033 mg/l/kg, *p* < 0.001) from 1.06 to 0.88 mg/l for males and from 1.04 to 0.87 mg/l for females. Following the second year of age, the levels remain stable for eight years. From 11 to 14 years of age, there is an increase of median CysC levels in males to 0.98 mg/l and a decrease in females to 0.86 mg/l. The change is associated with puberty (*ß* = 0.105 mg/l/Tanner stage, *p* < 0.001 in males and *ß* = − 0.093 mg/l/Tanner stage, *p* < 0.01 in females) and in males with height (*ß* = 0.003 mg/l/cm, *p* < 0.001).

**Conclusions:**

CysC levels depend on age, gender, and height, especially during infancy and puberty. We recommend the use of age- and gender-specific reference values for CysC serum levels for estimating kidney function in clinical practice.

**Electronic supplementary material:**

The online version of this article (10.1007/s00467-018-4087-z) contains supplementary material, which is available to authorized users.

## Introduction

Cystatin C (CysC), a cysteine protease inhibitor and low molecular weight protein, is an endogenous marker for glomerular filtration rate (GFR). Thus, the kidney function may be estimated based on CysC [1]. It is produced by all human nucleated cells at a stable rate, as it is the product of a housekeeping gene [2]. In contrast, creatinine is produced by the muscle tissue [3]. Therefore, the currently used GFR estimation formula (0.413 × height/sCrea(mg/dl)) must consider serum creatinine (sCrea) levels as well as the body height [4] due to varying body composition (especially muscle mass) causing inter- and intra-patient variability in sCrea levels [5–8]. Besides its glomerular filtration, creatinine is also secreted by the proximal tubules, leading to an overestimation of GFR, especially in patients with mild renal impairment [9]. CysC, however, is freely filtered in the glomeruli and demonstrates a high correlation with GFR determined by the gold standard such as CrEDTA [6, 10–12]. CysC levels are measured using the rapid and precise particle enhanced turbidimetric or nephelometric immunoassays (PETIA and PENIA) [13, 14].

Previously published data suggest that CysC allows the assessment of the renal function independently from age and gender, so a universal reference range of 0.63–1.08 for 1–18-year-old children was proposed (of note: before 2012/13, different calibrators were used in commercial assays having led to slightly different reference ranges) [15–18]. Nevertheless, newborns and infants show higher CysC levels [15–17, 19, 20] and reach steady levels after 1 to 3 years of age [7, 16, 19, 21]. Apparently independent of age and gender, the CysC levels remain constant up to the age of 14 to 16 years, according to some studies even up to adulthood [17]. Yata et al. (*n* = 1128) as well as Groesbeck et al. (*n* = 719) published the first larger pediatric cohort studies showing that CysC levels decrease in adolescents aged 15–16 years and are elevated in males compared to females at that same age [19, 22]. Miliku et al. found that GFR estimation equations using CysC were negatively associated with body mass index (BMI) and body surface area (BSA), but not lean or fat mass percentage [23]. An effect of age, gender, height, and weight on CysC was also found in adults [24].

This study aims to establish age- and gender-specific CysC reference values for generally healthy infants, children, and adolescents. Furthermore, we aim to analyze the effect of pubertal stage, height, weight, and BMI on CysC serum levels.

## Methods

### Design and study population

This article is structured according to the STROBE Checklist (Strengthening the Reporting of Observational Studies in Epidemiology) [25]. As part of the Leipzig Research Centre for Civilization Diseases (LIFE), the population-based cohort study LIFE Child has started recruiting urban, primarily healthy infants, children, and adolescents in Leipzig (Germany) in 2011. This large population-based cohort has already been used to establish reference intervals for serum lipids [26], liver enzymes [27], and iron-related blood parameters [28] in children. The examinations take place in the LIFE Child study center and are carried out by trained medical staff using highly standardized procedures [29, 30]. LIFE Child pursues the Declaration of Helsinki [31] and has been approved by the Ethics Committee of the University of Leipzig (Reg. No. 264-10-19042010). It is registered under the NCT trial number 02550236. All data were appropriately anonymized to comply to the German data protection law. More information including the recruitment process and repetitive examinations can be found in Poulain et al. and Quante et al. [29, 30].

**Fig. 1:**
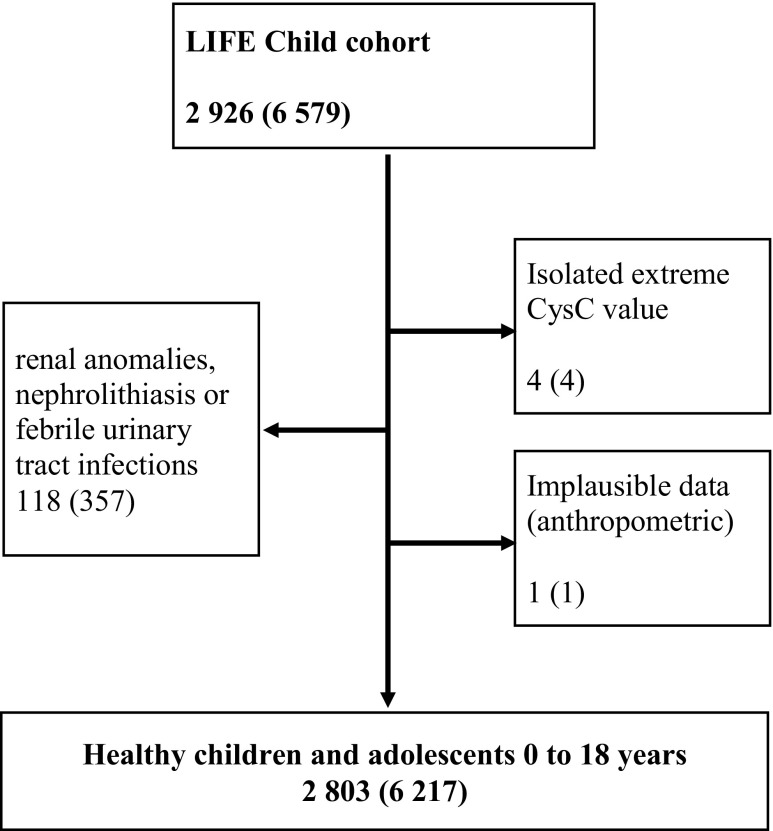
Flow chart for the participants of this study. Numbers of participants (observations). 2926 participants were observed at the age of 3, 6, and 12 months and thereafter up to once a year. We excluded participants with renal anomalies, nephrolithiasis, or febrile urinary tract infections. One participant was excluded due to implausible anthropometric data. Furthermore, isolated extreme values were excluded. In summary, 6217 observations of 2803 participants were available for analysis

In this study, all participants of the LIFE Child cohort having valid CysC measurements taken between 2011 and 2017 (2926 participants) were included. Children with an age of 0–16 years can participate in the study and receive invitations for follow-up examinations until the age of 18 years. Furthermore, during the first year of life, there are visits at the age of 3, 6, and 12 months. Thus, participants provided data on one to six follow-up visits. We excluded all participants with renal anomalies, nephrolithiasis, or febrile urinary tract infections (118 participants). This information was obtained through computer-assisted personal interview and sonography diagnostic. Furthermore, we identified and excluded four remaining isolated extreme values of CysC (< 0.4 mg/l) as well as one participant with implausible anthropometric data. Thus, a total of 6217 observations of 1337 females and 1466 males (age 0–18 years) are included in this study (Fig. 1).

### Laboratory assessment

We examined the CysC levels depending on age and gender. Furthermore, we examined sCrea levels in order to analyze whether or not our data is comparable to sCrea cohorts of earlier studies. For CysC and sCrea, morning venous blood was drawn from each participant by venipuncture using serum monovettes (Sarstedt AG&Co, Nümbrecht, Germany). The analyses were performed by the Institute for Laboratory Medicine, Clinical Chemistry and Molecular Diagnostics (ILM), University Hospital Leipzig. Serum samples were analyzed on an automated laboratory analyzer, Cobas8000 (Roche Diagnostics, Mannheim Germany), according to manufacturer’s protocol. sCrea was measured with an enzymatic assay (Roche Diagnostics). Measurement of serum CysC was performed using the turbidimetric immunoassay (PETIA) Tina-quant® Cystatin C (Roche Diagnostics). The primary measurement range is 0.4–8.0 mg/l. Traceability of the method was standardized against a Roche in-house reference preparation of recombinant human CysC. In April 2015, the Tina-quant® Cystatin C-assay was advanced to the second generation (*n* = 2070 during second versus *n* = 4401 observations during the first generation), now standardized against the international reference material ERM-DA471/IFCC [30]. Between October 2011 and April 2015, the variation coefficient of control level 1 varied between 1.3 and 6.4% (mean 3.0%), control level 2 varied between 0.9 and 4.5% (mean 2.0%). The primary measurement range of the Tina-quant® Cystatin C 2nd generation is 0.4–6.8 mg/l. Comparative measurement of 143 serum samples was performed between Tina-quant® Cystatin C and Tina-quant® Cystatin C 2nd generation. Using the MedCalc (MedCalc Software bvba, Belgium), a Passing-Bablok-regression [32] and Bland-Altman-plot [33] were calculated (Online Resources 2 and 3). The comparison showed a good conformity between the first and second generation of the immunoassay Tina-quant®. The mean bias accounts for 0.03 mg/l. The CysC reference values were not corrected for this clinically not relevant bias, which is also comparable to the usual batch effects.

### Anthropometric assessment

Height, weight, BMI, and puberty status were taken into account as potential confounding variables. BMI was calculated using height and weight measured by instructed and qualified personnel applying standardized procedures and regularly calibrated devices (a stadiometer with a measurement accuracy of 0.1 cm and a Seca 701 scale with a measurement accuracy of 50 g). The puberty status was examined by means of Tanner stages and assessed by trained staff members [34, 35].

### Statistics

**Fig. 2:**
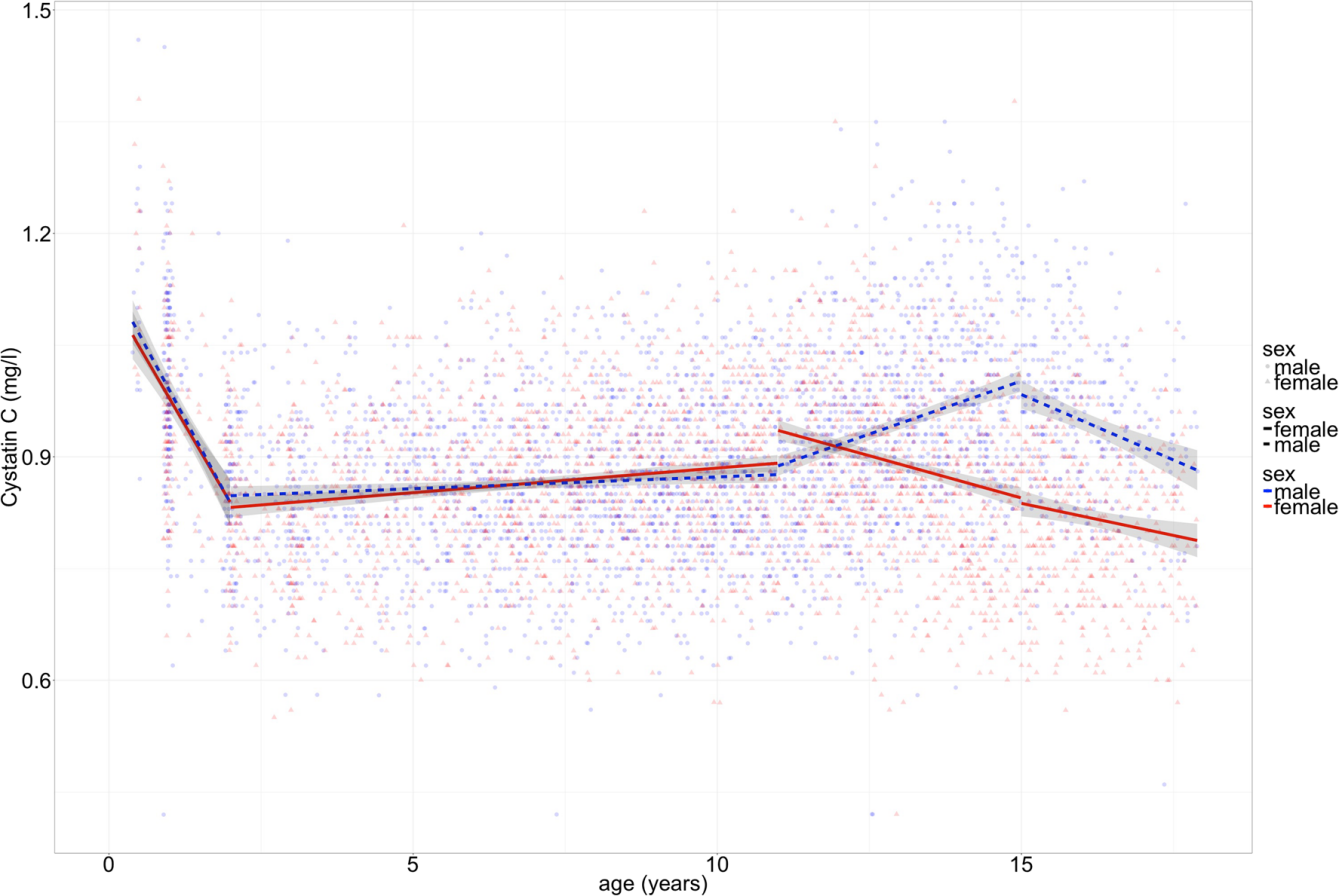
Cystatin C depends on age in infant and adolescent participants of the LIFE Child cohort. Solid line = female, dashed line = male participants. Note that at the age of 12 years, the curves diverge and show different patterns for males and females thereafter. *n* = 6217 observations of 2803 participants (0–18 years old)

The percentiles were estimated applying generalized additive models for location, shape, and scale as implemented in the gamlss package combined with a resampling method using the ChildSDS packages as described by Vogel et al. [36, 37]. All statistical analyses and visualization were done using the R-Software (version 3.3.2) [38]. To examine the influence of anthropometric measures on CysC levels, we stratified the data into four age intervals of linear course identified through visual inspection and local non-parametric regression (infancy 0–2 years, childhood 2–11 years, and adolescence with 11–15 and 15–18 years; see also Fig. 2 created with ggplot) [39]. Linear modeling was favored due to better interpretability. Hierarchical linear regression analyses (backward deletion) were applied to determine the effects of the independent variables on CysC levels (lmer-function of the R-package lme4) [40]. To account for repetitive measurements in follow-up participants, the subject was added as random effect on the intercept. *T* tests were used to compare mean CysC and sCrea levels of boys and girls (Table 1).

**Table 1:** Numbers of participants, observations, cystatin C (CysC) mean, intercept, and ß-slope of the LIFE Child cohort sorted in age intervals

		Infancy	Childhood	Adolescence		All
Age in years		0–2	2–11	11–15	15–18	0–18
Observations, *n* (%)	Males	213 (6)	1712 (52)	1002 (30)	363 (11)	3290 (100)
	Females	169 (6)	1433 (49)	895 (31)	430 (15)	2927 (100)
Participants	Males	180	903	540	245	1466
	Females	146	771	490	278	1337
CysC mean (±SD) in mg/l	Males	0.964 (0.149)	0.863 (0.106)	0.940 (0.128)	0.940 (0.117)	0.901 (0.124)
	Females	0.950 (0.134)	0.864 (0.105)	0.886 (0.123)	0.813 (0.108)	0.868 (0.117)
	*t* test	*p* > 0.05	*p* > 0.05	*p* < 0.001	*p* < 0.001	*p* < 0.001
CysC intercept in mg/l	Males	1.152	0.846	0.886	0.973	0.860
	Females	1.127	0.828	0.933	0.830	0.902
	*p* value	*p* > 0.05	*p* > 0.05	*p* < 0.001	*p* < 0.01	*p* < 0.001
CysC ß-slope in mg/l/a	Males	− 0.159	0.003	0.028	− 0.033	0.005
	Females	− 0.148	0.007	− 0.023	− 0.017	− 0.003
	*p* value	*p* > 0.05	*p* < 0.05	*p* < 0.001	*p* > 0.05	*p* < 0.001
Height mean (±SD) in cm	Males	78.2 (5.77)	124.9 (18.0)	161.4 (11.5)	176.6 (7.1)	138.7 (29.4)
	Females	76.3 (5.9)	123.5 (18.3)	159.7 (8.6)	166.5 (6.3)	138.2 (27.9)
Weight mean (±SD) in kg	Males	10.19 (1.67)	27.03 (10.91)	54.21 (16.10)	68.47 (16.78)	38.79 (21.59)
	Females	9.45 (1.68)	26.51 (11.21)	53.93 (15.27)	62.74 (15.41)	39.20 (21.11)
BMI mean (±SD) in kg/m^2^	Males	16.6 (1.2)	16.7 (3.0)	20.5 (4.6)	21.9 (4.8)	18.4 (4.3)
	Females	16.0 (1.1)	16.7 (3.1)	21.0 (5.0)	22.6 (5.3)	18.8 (4.8)

Age intervals were determined by visual inspection for better interpretation with linear regression models (see Fig. *2*). Note that participants may have undergone various observations that belong to different age intervals. Intercepts and ß-slopes (in mg/l per year of life) were calculated with the lm-function in R. The intercept represents the mean CysC concentration of the first observations of each age interval

*n* number of observations, *SD* standard deviation, *BMI* body mass index

## Results

A total of 2803 participants with 6217 observations (Fig. 1 and Table 1) were included. The distributions of pubertal stages and BMI in the LIFE Child cohort are summarized in Table 2.

**Table 2:** Distribution of puberty status and BMI in the LIFE Child cohort (0–18 years)

	Puberty status (Tanner stage)						BMI				
	1	2	3	4	5	All	Underweight	Normal	Overweight	Obese	All
Males, *n* (%)	1541 (67)	332 (14)	121 (5)	172 (7)	149 (6)	2315 (100)	244 (7)	2529 (77)	203 (6)	288 (9)	3264 (100)
Males age mean (±SD) in years	6.5 (3.1)	11.6 (1.2)	13.0 (1.2)	14.2 (1.2)	15.9 (1.6)	9.5 (4.4)					
Females, *n* (%)	1324 (50)	302 (11)	269 (10)	292 (11)	441 (17)	2628 (100)	214 (7)	2194 (76)	199 (7)	284 (10)	2891 (100)
Females age mean (±SD) in years	5.9 (2.9)	10.9 (1.2)	12.4 (1.3)	14.2 (1.6)	15.9 (1.7)	9.9 (4.6)					

Puberty status: pre-pubertal (Tanner stage 1), pubertal (Tanner stages 2–4), post-pubertal (Tanner stage 5). BMI groups: underweight < 10th percentile, overweight > 90th percentile, obese > 97th percentile. As the puberty status and BMI were not examined in all participants, the total numbers of all observations and participants in this table differ from the numbers presented in Fig. 1

*n* number of observations, *BMI* body mass index

### Serum creatinine distribution

The percentiles of sCrea levels for girls and boys are provided in the Online Resources 1 and 4. First, we evaluated the sCrea distribution to show that the LIFE Child cohort is comparable to other studies and, therefore, a representative sample of the population; sCrea levels rise continuously until the age of 12.5 years (ß-slope = 2.744 μmol/l/a = 0.031 mg/dl/a; a = period of 1 year of life) for both boys and girls. At that age, median sCrea levels are 53 μmol/l = 0.60 mg/dl in girls and 55 μmol/l = 0.62 mg/dl in boys. sCrea levels increase more rapidly in 12.5- to 18-year-old boys (*ß* = 5.905 μmol/l/a = 0.067 mg/dl/a). In contrast, the slope (ß) in 12.5- to 18-year-old girls is constant at around 2.8 μmol/l/a = 0.032 mg/dl/a. From the age of 13 years, boys exhibit significantly (*p* < 0.01) higher sCrea levels than girls.

### Serum cystatin C distribution

**Fig. 3:**
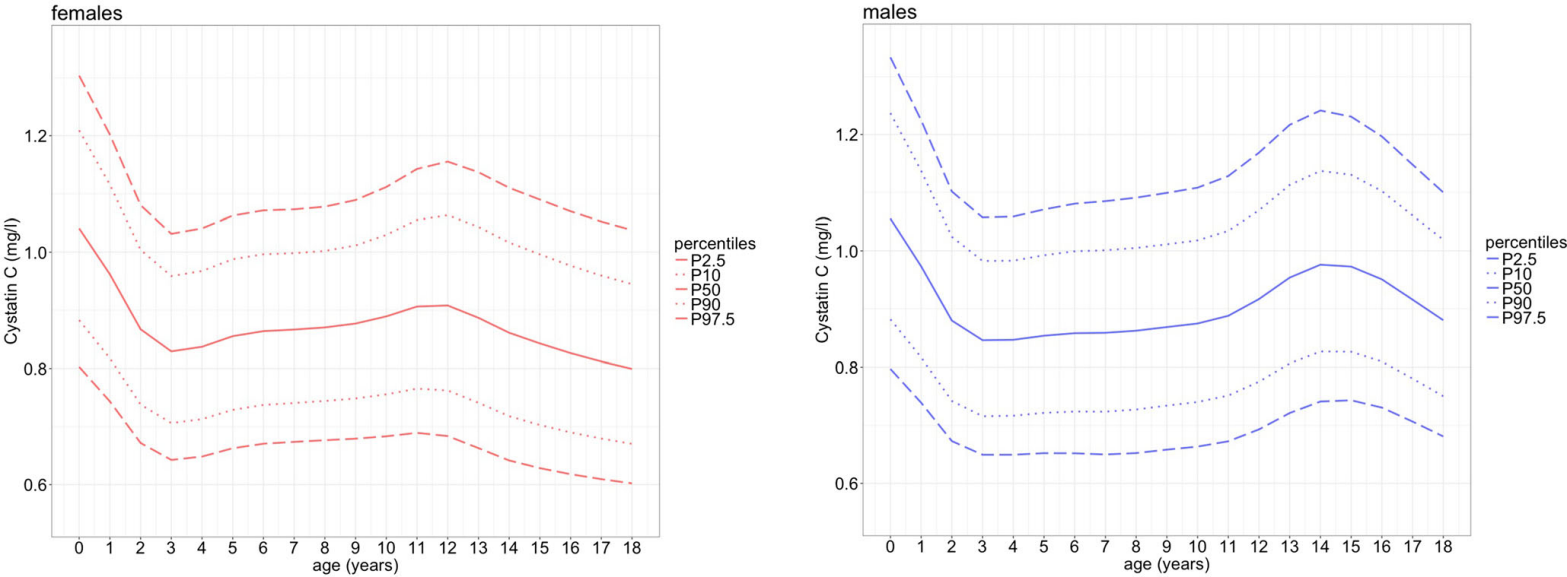
Percentiles of cystatin C and its effector variable age for 0- to 18-year-old children of the LIFE Child cohort. Solid line = 50th percentile, dotted line = 10th and 90th percentile, dashed line = 2.5th and 97.5th percentile. P percentile. The percentiles were calculated using the ChildSDS package [37]. Note that just before the age of 12 years, the curves diverge and show different patterns for males and females thereafter. *n* = 6217 observations in 2803 participants

The distribution of CysC levels in the LIFE Child cohort, the percentiles and the degree of freedom spread, skewness, location, and kurtosis parameters are shown in Fig. 3 and Table 3. Measurements were elevated for children between the age of 3 months and 18 years. The median CysC serum concentrations are highest in toddlers (males 1.06 mg/l, females 1.04 mg/l). They decrease during the first 2 years of life (*ß* = − 0.154 mg/l/a) to slightly but significantly lower levels (*p* < 0.001; males: 0.88 mg/l; females: 0.87 mg/l) and remain constant during childhood until the age of 11 years. The mean CysC values in girls and boys do not differ significantly at this age (*p* > 0.05). While the serum levels of female adolescents start to decrease at 11 years (*ß* = − 0.023 mg/l/a), those for male adolescents increase (*ß* = 0.028 mg/l/a). Thus, at the age of 13 years, CysC levels differ significantly between males and females (*p* < 0.001). After reaching 15 years of age and median levels of 0.97 mg/l in males and 0.84 mg/l in females, CysC levels of male participants drop again (*ß* = − 0.033 mg/l/a). In our study cohort, we found that CysC levels in males and females remained significantly different until the age of 18 (*p* < 0.05) with the highest and most significant difference at the age of 15 years (*p* < 0.001, mean CysC levels 0.97 mg/l in males and 0.84 mg/l in females).

**Table 3:** (a) Percentiles of cystatin C (mg/l) as a function of age based on the LIFE Child cohort with 0- to 18-year-old boys. (b) Percentiles of cystatin C (mg/l) as a function of age based on the LIFE Child cohort with 0- to 18-year-old girls

Age	*n*	P2.5	P5	P10	P50	P90	P95	P97.5	Mu	Sigma	Nu	Tau
(a)												
0	106	0.80	0.84	0.88	1.06	1.24	1.29	1.33	1.06	0.13	0.73	2.12
1	107	0.74	0.77	0.82	0.97	1.14	1.18	1.22	0.97	0.13	0.73	2.09
2	135	0.67	0.70	0.74	0.88	1.02	1.07	1.10	0.88	0.12	0.72	2.05
3	132	0.65	0.68	0.72	0.85	0.98	1.02	1.06	0.85	0.12	0.71	2.00
4	141	0.65	0.68	0.72	0.85	0.98	1.02	1.06	0.85	0.12	0.71	1.96
5	156	0.65	0.68	0.72	0.85	0.99	1.03	1.07	0.85	0.12	0.70	1.90
6	198	0.65	0.69	0.72	0.86	1.00	1.04	1.08	0.86	0.13	0.70	1.85
7	197	0.65	0.68	0.72	0.86	1.00	1.05	1.09	0.86	0.13	0.69	1.79
8	251	0.65	0.69	0.73	0.86	1.01	1.05	1.09	0.86	0.13	0.67	1.74
9	260	0.66	0.69	0.73	0.87	1.01	1.06	1.10	0.87	0.13	0.64	1.69
10	242	0.66	0.70	0.74	0.87	1.02	1.07	1.11	0.87	0.13	0.61	1.65
11	265	0.67	0.71	0.75	0.89	1.03	1.08	1.13	0.89	0.13	0.58	1.61
12	259	0.69	0.73	0.78	0.92	1.07	1.12	1.17	0.92	0.13	0.55	1.59
13	253	0.72	0.76	0.81	0.95	1.11	1.17	1.22	0.95	0.13	0.54	1.59
14	225	0.74	0.78	0.83	0.98	1.14	1.19	1.24	0.98	0.13	0.53	1.61
15	190	0.74	0.78	0.83	0.97	1.13	1.18	1.23	0.97	0.12	0.54	1.65
16	110	0.73	0.77	0.81	0.95	1.10	1.15	1.20	0.95	0.12	0.56	1.69
17	63	0.71	0.74	0.78	0.92	1.06	1.11	1.15	0.92	0.12	0.58	1.76
(b)												
0	86	0.80	0.84	0.88	1.04	1.21	1.26	1.30	1.04	0.12	0.59	1.97
1	83	0.74	0.78	0.82	0.96	1.12	1.16	1.20	0.96	0.12	0.61	1.96
2	115	0.67	0.70	0.74	0.87	1.00	1.04	1.08	0.87	0.12	0.63	1.95
3	110	0.64	0.67	0.71	0.83	0.96	1.00	1.03	0.83	0.12	0.65	1.94
4	119	0.65	0.68	0.71	0.84	0.97	1.01	1.04	0.84	0.12	0.67	1.93
5	139	0.66	0.69	0.73	0.86	0.99	1.03	1.06	0.86	0.12	0.69	1.91
6	183	0.67	0.70	0.74	0.86	1.00	1.04	1.07	0.86	0.12	0.70	1.89
7	181	0.67	0.70	0.74	0.87	1.00	1.04	1.07	0.87	0.12	0.70	1.87
8	181	0.68	0.71	0.74	0.87	1.00	1.04	1.08	0.87	0.12	0.70	1.85
9	201	0.68	0.71	0.75	0.88	1.01	1.05	1.09	0.88	0.12	0.70	1.83
10	204	0.68	0.72	0.76	0.89	1.03	1.07	1.11	0.89	0.12	0.68	1.82
11	214	0.69	0.72	0.77	0.91	1.06	1.10	1.14	0.91	0.13	0.66	1.80
12	235	0.68	0.72	0.76	0.91	1.06	1.11	1.16	0.91	0.13	0.63	1.80
13	224	0.66	0.70	0.74	0.89	1.04	1.09	1.14	0.89	0.13	0.58	1.79
14	222	0.64	0.68	0.72	0.86	1.02	1.07	1.11	0.86	0.14	0.53	1.78
15	188	0.63	0.66	0.70	0.84	1.00	1.05	1.09	0.84	0.14	0.48	1.77
16	151	0.62	0.65	0.69	0.83	0.98	1.03	1.07	0.83	0.14	0.42	1.76
17	91	0.61	0.64	0.68	0.81	0.96	1.01	1.05	0.81	0.14	0.36	1.74

The 2.5th, 10th, 90th, and 97.5th percentiles as well as the median are given

*n* participants, *P* percentile, *Mu* location parameter, *Sigma* spread parameter, *Nu* skewness parameter, *Tau* kurtosis parameter

Among all participants of the LIFE Child cohort, the scale remains constant as indicated by the sigma-value of 0.12–0.14 mg/l (Table 3).

### Effects of height, weight, BMI, puberty, and age on cystatin C

To identify potential influential factors for the changes in CysC levels during infancy and adolescence, we correlated height, weight, BMI, the Tanner stage, and age with the CysC concentrations for boys and girls separately. For better interpretability, linear regression models were applied to four different intervals of linear course identified through visual inspection (infancy 0–2 years, childhood 2–11 years, adolescence with 11–15 and 15–18 years; Fig. 2 and Table 1). All effects are corrected for age (except age itself) and repetitive measurement in follow-up participants.

In a simple linear regression analysis of the participants aged 0–2 years, age, height, and weight were shown to be negatively associated with CysC serum concentrations (*p* < 0.001), whereas BMI does not show a significant effect. In hierarchical regression analyses, CysC levels were negatively correlated with height (*ß* = − 0.010 mg/l/cm, *p* < 0.001) as well as weight (*ß* = − 0.033 mg/l/kg, *p* < 0.001).

Using stepwise multiple regression models including age, height, weight, BMI, and Tanner stages CysC levels of male participants between the age of 11 and 14 years showed a strong dependency on puberty status (*p* < 0.001). Even after adjustment for age, CysC concentrations are significantly higher in pubertal boys (especially in Tanner stages three and four with *ß* ≈ 0.1 mg/l/Tanner stage, *p* < 0.001), compared to prepubertal boys. In females at the same age, pubertal stage is also the strongest predictor of CysC levels (*p* < 0.001), but in contrast to males negatively associated with CysC levels (*ß* = − 0.093 mg/l/Tanner stage, *p* < 0.01). We observe a peak of CysC concentrations in female adolescents during pubertal stage two that however is not significantly different from prepubertal CysC concentrations. Weight and BMI show no significant effect on CysC levels in adolescents. Height is another predictor of serum CysC concentrations in pubertal males (*ß* = 0.003 mg/l/cm, *p* < 0.001).

## Discussion

This study aimed to propose CysC reference values for healthy infants, children, and adolescents. We have shown that our study cohort is a representative sample as sCrea levels are distributed similar to published results from earlier population-based studies [16, 41–43]. CysC levels depend on height, weight, age, and puberty. In newborns, CysC levels are higher than at later ages. They decrease rapidly during the first 2 years of life, being negatively associated with height and weight. In 11- to 14-year-old adolescents, the puberty status is the strongest predictor of CysC serum concentrations with an increase of CysC levels in males during early puberty and a decrease in females during late puberty.

Miliku et al. recently published the results of a study in healthy 6-year-olds in Rotterdam (Netherlands) using the same Roche kit for CysC analysis. They did not find significant associations of sex and CysC levels nor the eGFR (calculated using CysC levels with the Zappitelli formula) and sex. Puberty was not considered as they analyzed values of an age-homogeneous population without newborns and pubertal adolescents [23].

The strength of our study is a broad age range from 0 to 18 years and a large number of observations (*n* = 6217) of healthy participants and a standardized assessment. To our knowledge, LIFE Child is the first European study to present data from such a large cohort including very young infants from the age of 3 months. Nevertheless, the results are based on the social distribution in Leipzig [29, 30]. Therefore, cohort studies in other geographical areas such as Marmarinos et al. may be necessary in order to take regional variability into account [44].

Although earlier studies used different measuring methods for CysC, the course of the percentiles can be compared: The given percentiles for CysC levels are concordant to those proposed by earlier studies (0.63–1.08 mg/l) [15–18, 45], but do not support the thesis of age- and gender-independent reference values. We found that infants exhibited higher CysC levels up to the age of 2 years, thereby confirming the results of other studies such as Andersen et al., Ridefelt et al., Randers et al., and Filler et al. [15–17, 19–21]. A possible explanation is the maturation of kidney function: only the juxtamedullary glomeruli filter blood in newborns, while all other nephrons—although already terminally differentiated—are recruited up to the age of 18–24 months [46, 47]. In 11- to 14-year-old male adolescents, the median CysC concentrations increase to about 0.98 mg/l and thereafter constantly drop to mean values of 0.88 mg/l. In female adolescents, these parameters are up to 0.13 mg/dl lower. This partly confirms the percentiles described by Yata et al. (Japan), Groesbeck et al. (USA), and Marmarinos et al., who were the first to conduct larger pediatric cohort studies (*n* = 1128, 719, and 536, respectively) and showed that CysC levels depend on age and gender during adolescence [19, 22, 44].

In contrary to the results of Marmarinos et al., the BMI shows no significant effect on CysC that cannot be explained by the single variables height or weight. The low correlation coefficient of *r*^2^ = 0.003 (*p* < 0.001) may explain why no correlation with lean or fat mass percentage was found by Miliku et al. in 6-year-old children [23, 44]. The estimation of the GFR based on sCrea must also consider the body height (Schwartz et al.) [4] during entire childhood and adolescence. CysC shows small variance due to height in 0- to 2-year-old infants (*ß* = − 0.010 mg/l/cm) and 11- to 14-year-old male adolescents (*ß* = 0.003 mg/l/cm). Thus, body growth may affect CysC concentrations as supposed by its association with height in infancy and male adolescents during puberty. The hypothesis is that during body growth more body cells exist and so more housekeeping protein CysC will be produced. That leads to a rise in CysC concentrations, which appears especially applicable to pubertal boys due to a higher body growth compared to pubertal girls.

We found an increase of CysC levels in male and a decrease in female adolescents associated with pubertal development. There is no explanation so far, why pubertal development has a reverse effect on CysC serum concentrations in male and female adolescents. Similar to the association described by Groesbeck et al., CysC levels of females showed a peak in Tanner stage two whereas those of male participants had a peak in Tanner stage four [22]. At the age of 13 years, the CysC levels start to be significantly different for males and females. At the age of 15 years, this difference amounts to 0.13 mg/l (15.5% higher in males compared to females, Table 1) and is similar to that of sCrea levels at the same age (15.2% higher in males compared to females). We consider this difference as clinically relevant.

In clinical practice, kidney injury is diagnosed by loss of estimated GFR or increase in sCrea by 25%, which depends highly on muscle mass [48]. Any known gender- or age-related changes in parameters of normal kidney function are necessary for the recognition of renal damage. This especially applies to formerly unknown patients at the time of admission for example onto a pediatric intensive care unit. Therefore, when using CysC parameters, we suggest the use of age- and gender-related CysC reference values to evaluate renal function in pediatric patients.

Overall, growth rate, serum levels of sexual hormones, blood glucose, smoking or alcohol consumption may affect CysC serum concentrations. As we continue our research, we aim to include the socioeconomic status among the other potential effector variables and confounders in subsequent studies.

Nevertheless, the percentiles of this study suggest that CysC serum concentration is a stable parameter with narrow ranges, but with a notable variation in infancy and adolescence related to age, gender, and puberty.

## Conclusion

Our study provides CysC reference values derived from a large pediatric cohort in a homogeneous Caucasian population (6217 observations of 2803 participants). The results of this population-based cohort indicate that serum CysC levels do vary significantly according to age, gender, and pubertal status. Therefore, we suggest the use of age and gender-specific reference ranges for the assessment of kidney function in newborns, children, and adolescents.

## Electronic supplementary material

ESM1 (PDF 292 kb)
