# Supplementary material for: Cystatin C serum levels in healthy children are related to age, gender, and pubertal stage
Source: Pediatr Nephrol. 2018 Nov 20;34(3):449–57. doi: 10.1007/s00467-018-4087-z (PMC6349798; doi:10.1007/s00467-018-4087-z)
Supplement: Supplementary file 1 — (PDF 292 kb) [file 467_2018_4087_MOESM1_ESM.pdf]

# Supplementary Materials

# Cystatin C serum levels in healthy children are related to age, gender and pubertal stage

Niels Ziegelasch[1], Mandy Vogel[1,2], Markus Löffler, MD [3], Eva Müller, MD[1], Nadin Tremel, MD[1], Anne Jurkutat[1], Nicolas Terliesner, MD[4], Joachim Thiery, MD[5], Anja Willenberg, PhD[5], Wieland Kiess, MD[1,2,4], Katalin Dittrich, MD[2,4]

[1] LIFE Leipzig Research Centre for Civilization Diseases, University of Leipzig, Philipp-Rosenthalstrasse 27, 04103 Leipzig, Germany

[2] Centre of Paediatric Research (CPL), University of Leipzig, 04103 Leipzig, Germany

[3] Institute for Medical Informatics, Statistics and Epidemiology (IMISE), University of Leipzig, 04107 Leipzig

[4] Hospital for Children and Adolescents, University of Leipzig, 04103 Leipzig, Germany

[5] Institute for Laboratory Medicine, Clinical Chemistry and Molecular Diagnostic (ILM), University Hospital Leipzig, 04103 Leipzig, Germany

**Corresponding Author:** Dr. med. Katalin Dittrich, Hospital for Children and Adolescents, University of Leipzig, Liebigstraße 20a, D-04103 Leipzig; Phone: +49 341 9726325 Fax: +49 341 9726009, E-Mail: katalin.dittrich@medizin.uni-leipzig.de

**Online Resource 1.** Percentiles of screa and its effector variable age for 0- to 18-year-old-children of the LIFE Child cohort.

**Online Resource 2.** Passing-Bablok Regression Cystatin C 2nd generation against 1st Generation.

**Online Resource 3.** Bland-Altman-Blot absolute deviation.

**Online Resource 4.** Percentiles of sCrea (µmol/l) as a function of age based on the LIFE Child cohort with 0- to 18-year-old children.

**Online Resource 1.** Percentiles of sCrea and its effector variable age for 0- to 18-year-old-children of the LIFE Child cohort.

Blue = male, red = female participants. Solid line = 50th percentile, dotted line = 10th and 90th percentile, dashed line = 2.5th and 97.5th percentile. P = Percentile. The percentiles were calculated using the ChildSDS package[36]. Note that at the age of 12 years, the curves diverge and show different patterns for males and females thereafter. n = 6 217 observations in 2 803 participants.

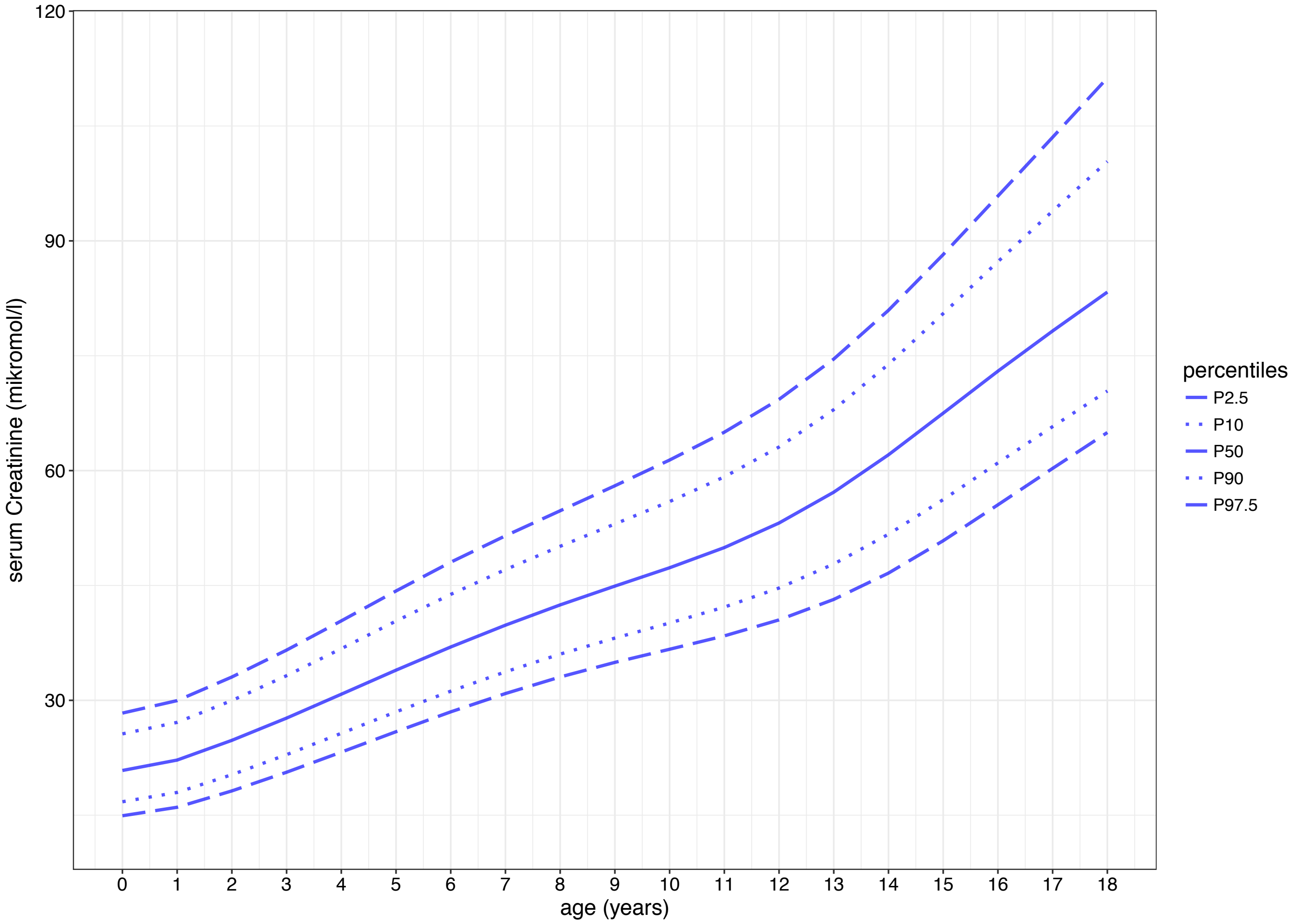

serum Creatinine (mikromol/l)
120
90
60
30
0
1
2
3
4
5
6
7
8
9
10
11
12
13
14
15
16
17
18
age (years)
percentiles
P2.5
P10
P50
P90
P97.5


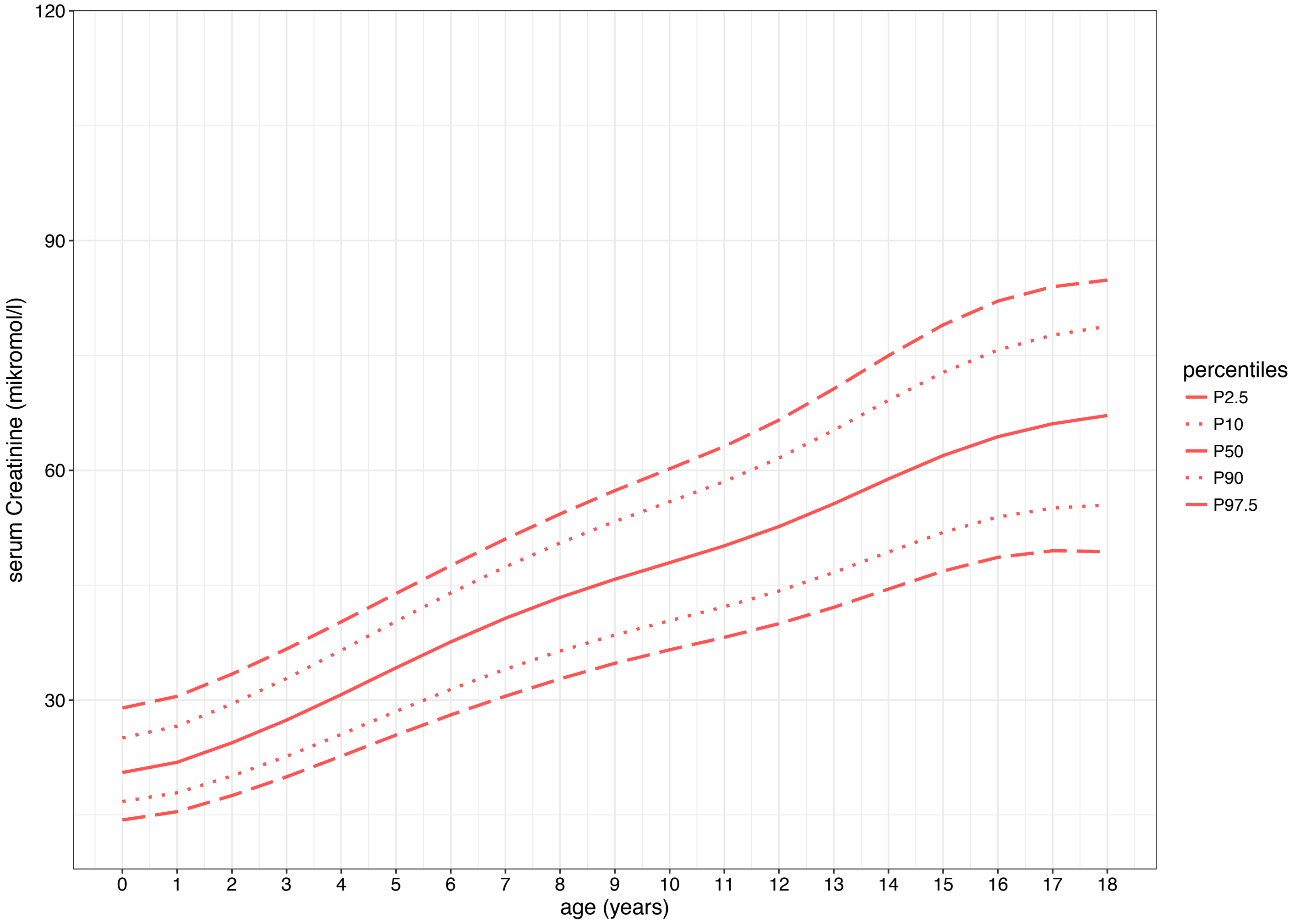

serum Creatinine (mikromol/l)
120
90
60
30
0
1
2
3
4
5
6
7
8
9
10
11
12
13
14
15
16
17
18
age (years)
percentiles
P2.5
P10
P50
P90
P97.5

**Online Resource 2**. Passing-Bablok Regression Cystatin C 2nd generation against 1st Generation

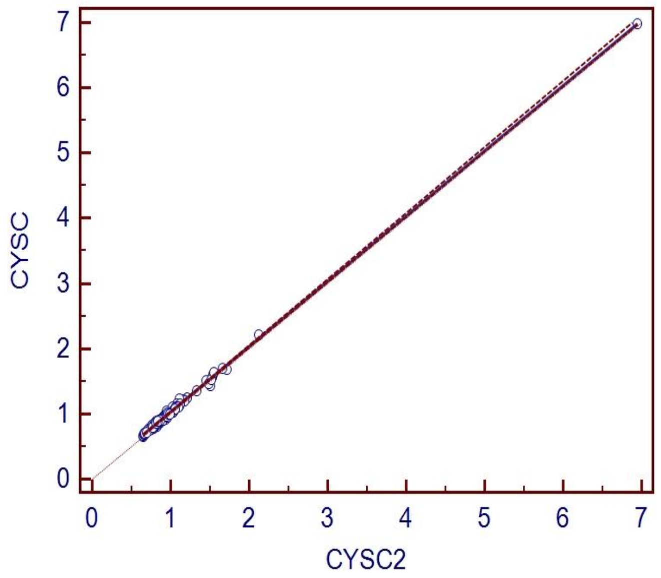


**Online Resource 3.** Bland-Altman-Blot absolute deviation

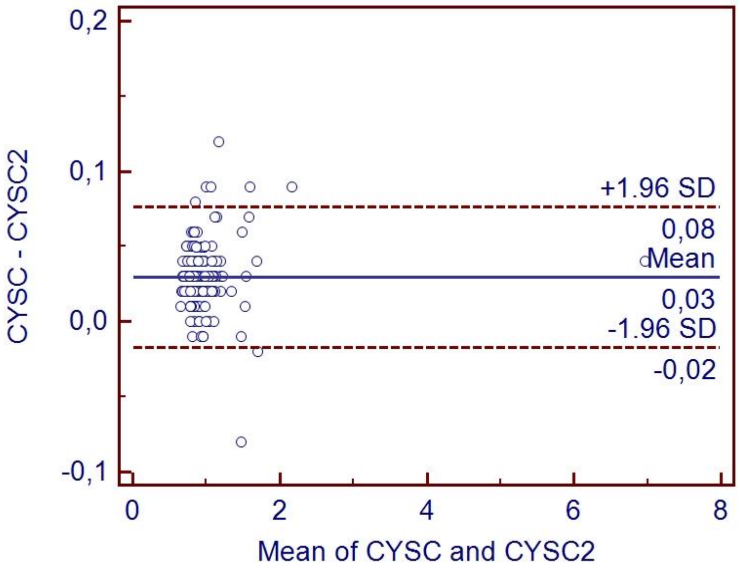


Comparative measurement of 143 serum samples between Tina-quant® Cystatin C and Tina-quant® Cystatin C 2nd generation. Using MedCalc (MedCalc Software bvba, Belgium) Passing-Bablok-regression (2) (Online Resource 2) and Bland-Altman-Blot (Online Resource 3) were calculated. Both show a good conformity between the first and the second generation.

**Online Resource 4.** *Percentiles of sCrea (µmol/l) as a function of age based on the LIFE Child cohort with 0- to 18-year-old children.*

| age | P2.5 M | P2.5 F | P10 M | P10 F | P50 M | P50 F | P90 M | P90 F | P97.5 M | P97.5 F | Mu M | Mu F | Sigma M | Sigma F | Nu M | Nu F | Tau M | Tau F |
|---|---|---|---|---|---|---|---|---|---|---|---|---|---|---|---|---|---|---|
| 0 | 14.92 | 14.34 | 16.75 | 16.76 | 20.84 | 20.54 | 25.62 | 25.06 | 28.33 | 28.97 | 20.84 | 20.54 | 0.16 | 0.17 | 0.26 | 0.13 | 2.14 | 1.21 |
| 1 | 16.04 | 15.43 | 17.97 | 17.89 | 22.20 | 21.88 | 27.12 | 26.59 | 29.96 | 30.49 | 22.20 | 21.88 | 0.16 | 0.16 | 0.26 | 0.15 | 2.08 | 1.28 |
| 2 | 18.17 | 17.53 | 20.29 | 20.09 | 24.77 | 24.42 | 29.97 | 29.50 | 33.05 | 33.39 | 24.77 | 24.42 | 0.15 | 0.16 | 0.24 | 0.18 | 1.99 | 1.40 |
| 3 | 20.61 | 19.98 | 22.90 | 22.65 | 27.68 | 27.39 | 33.22 | 32.82 | 36.56 | 36.67 | 27.68 | 27.39 | 0.15 | 0.15 | 0.20 | 0.25 | 1.94 | 1.52 |
| 4 | 23.24 | 22.68 | 25.69 | 25.53 | 30.80 | 30.72 | 36.75 | 36.48 | 40.38 | 40.22 | 30.80 | 30.72 | 0.14 | 0.14 | 0.14 | 0.41 | 1.92 | 1.66 |
| 5 | 25.90 | 25.44 | 28.49 | 28.52 | 33.94 | 34.21 | 40.35 | 40.28 | 44.28 | 43.91 | 33.94 | 34.21 | 0.14 | 0.14 | 0.06 | 0.63 | 1.93 | 1.78 |
| 6 | 28.49 | 28.07 | 31.20 | 31.40 | 36.97 | 37.60 | 43.83 | 43.98 | 48.04 | 47.56 | 36.97 | 37.60 | 0.13 | 0.13 | -0.03 | 0.83 | 1.96 | 1.89 |
| 7 | 30.89 | 30.51 | 33.72 | 34.04 | 39.81 | 40.68 | 47.07 | 47.41 | 51.48 | 51.03 | 39.81 | 40.68 | 0.13 | 0.13 | -0.05 | 0.93 | 1.99 | 1.96 |
| 8 | 33.05 | 32.78 | 36.03 | 36.41 | 42.46 | 43.41 | 50.11 | 50.52 | 54.76 | 54.31 | 42.46 | 43.41 | 0.13 | 0.13 | -0.06 | 0.90 | 1.99 | 2.02 |
| 9 | 34.95 | 34.81 | 38.13 | 38.50 | 44.91 | 45.78 | 53.03 | 53.32 | 58.02 | 57.35 | 44.91 | 45.78 | 0.13 | 0.13 | -0.08 | 0.79 | 1.96 | 2.04 |
| 10 | 36.67 | 36.56 | 40.10 | 40.37 | 47.31 | 47.94 | 55.96 | 55.90 | 61.38 | 60.20 | 47.31 | 47.94 | 0.13 | 0.13 | -0.08 | 0.70 | 1.91 | 2.04 |
| 11 | 38.41 | 38.19 | 42.17 | 42.19 | 49.95 | 50.15 | 59.18 | 58.54 | 65.02 | 63.12 | 49.95 | 50.15 | 0.13 | 0.13 | -0.02 | 0.67 | 1.87 | 2.02 |
| 12 | 40.50 | 39.99 | 44.67 | 44.24 | 53.15 | 52.67 | 63.08 | 61.61 | 69.30 | 66.57 | 53.15 | 52.67 | 0.14 | 0.13 | 0.09 | 0.64 | 1.86 | 1.99 |
| 13 | 43.18 | 42.11 | 47.80 | 46.66 | 57.18 | 55.64 | 67.96 | 65.24 | 74.57 | 70.64 | 57.18 | 55.64 | 0.14 | 0.13 | 0.21 | 0.60 | 1.88 | 1.96 |
| 14 | 46.61 | 44.50 | 51.69 | 49.33 | 62.06 | 58.88 | 73.87 | 69.15 | 80.94 | 74.98 | 62.06 | 58.88 | 0.14 | 0.13 | 0.27 | 0.56 | 1.92 | 1.95 |
| 15 | 50.84 | 46.85 | 56.21 | 51.90 | 67.50 | 61.94 | 80.51 | 72.81 | 88.21 | 79.00 | 67.50 | 61.94 | 0.14 | 0.13 | 0.21 | 0.53 | 1.98 | 1.95 |
| 16 | 55.53 | 48.65 | 61.01 | 53.91 | 72.99 | 64.40 | 87.29 | 75.72 | 95.85 | 82.11 | 72.99 | 64.40 | 0.14 | 0.13 | 0.01 | 0.55 | 2.03 | 1.97 |
| 17 | 60.29 | 49.50 | 65.74 | 55.07 | 78.24 | 66.09 | 93.89 | 77.66 | 103.53 | 83.98 | 78.24 | 66.09 | 0.14 | 0.13 | -0.27 | 0.71 | 2.08 | 2.01 |

The 2.5th, 10th, 90th, and 97.5th percentiles as well as the median are given. Blue = male, red = female participants. Abbreviations: P = percentile, M = male, F = female, Mu = location parameter, Sigma = spread Parameter, Nu = skewness parameter, Tau = kurtosis parameter. All values were calculated applying generalized additive models for location, shape and scale as implemented in gamlss [35] and childsds [36]. n = 6 217 observations in 2 803 participants. Note, that at the age of 12 years, sCrea values of male adolescents (P50) raise and are higher compared to females up to the age of 18 years.
